# Clinically abnormal case with paternally derived partial trisomy 8p23.3 to 8p12 including maternal isodisomy of 8p23.3: a case report

**DOI:** 10.1186/1755-8166-2-14

**Published:** 2009-06-30

**Authors:** Dilek Aktas, Anja Weise, Eda Utine, Dursun Alehan, Kristin Mrasek, Ferdinand von Eggeling, Heike Thieme, Ergul Tuncbilek, Thomas Liehr

**Affiliations:** 1 Hacettepe University Faculty of Medicine, Department of Genetics, 06100 Sihhiye, Ankara, Turkey; 2 Institut für Humangenetik und Anthropologie, Kollegiengasse 10, D-07743 Jena, Germany; 3 Hacettepe University Faculty of Medicine, Department of Peditarics Cardiology, 06100 Sihhiye, Ankara, Turkey

## Abstract

**Background:**

Because of low copy repeats (LCRs) and common inversion polymorphisms, the human chromosome 8p is prone to a number of recurrent rearrangements. Each of these rearrangements is associated with several phenotypic features. We report on a patient with various clinical malformations and developmental delay in connection with an inverted duplication event, involving chromosome 8p.

**Methods:**

Chromosome analysis, multicolor banding analysis (MCB), extensive fluorescence in situ hybridization (FISH) analysis and microsatellite analysis were performed.

**Results:**

The karyotype was characterized in detail by multicolor banding (MCB), subtelomeric and centromere-near probes as 46,XY,dup(8)(pter->p23.3::p12->p23.3::p23.3->qter). Additionally, microsatellite analysis revealed the paternal origin of the duplication and gave hints for a mitotic recombination involving about 6 MB in 8p23.3.

**Conclusion:**

A comprehensive analysis of the derivative chromosome 8 suggested a previously unreported mechanism of formation, which included an early mitotic aberration leading to maternal isodisomy, followed by an inverted duplication of the 8p12p23.3 region.

## Background

To date, a number of patients with inverted duplication of 8p have been identified through cytogenetic analysis [1-7] and different breakpoints related to 8p have been reported [4]. The distal breakpoint was predominantly in 8p23 and was found in combination with various proximal breakpoints (centromere, p11 and p12), but predominantly within 8p11.

An inverted duplication of 8p is associated with mental retardation, distinct facial anomalies, agenesis of corpus callosum and hypotonia. Although less common, congenital heart defects, coloboma, scoliosis and seizures are noted.

We report another patient with a complex rearrangement leading to an inverted duplication of 8p23.3 to 8p12. Phenotypic findings in our patient and previously reported chromosome 8p inverted duplications are reviewed and several important features are highlighted.

## Case presentation

### Clinical details

The male infant was the second child born to a non-consanguineous couple. Following a normal gestation and delivery, the boy was born at 40-weeks of gestation with a birth weight of 3.2 kg. There were no neonatal problems or feeding difficulty.

**Figure 1:**
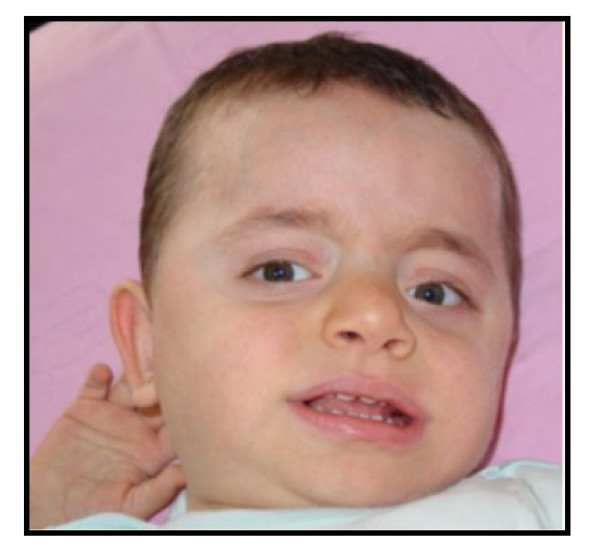
**Frontal view of the reported case at 15 months of age**.

At 15 months of age, his weight was 11.3 kg (10th–25th centiles), length was 87 cm (90th–95th centiles), and head circumference was 48 cm (10th–25th centiles). He was evaluated for motor and language delay. Dysmorphic facial features including brachycephaly, prominent forehead, prominent nasal bridge, flared alae nasi, wide mouth with thin upper lip were present (Figure 1). Ears were large and posteriorly rotated. He had no eye or skeletal abnormalities. There was mild generalized hypotonia. He was still unable to sit and walk. He could roll on both sides, transfer objects hand-to-hand, but he could not use a spoon or fork for self-feeding.

An abdominal ultrasound was normal. Two-dimensional echocardiogram revealed small muscular ventricular septal defect. Brain MRI demonstrated agenesis of corpus callosum.

## Methods

Karyotyping was performed on metaphase spreads prepared from peripheral blood lymphocytes by conventional methods. The aberrant karyotype was further studied applying multicolor banding (MCB) probe sets for chromosome 8 [8]; MCB-results were evaluated using the software of MetaSystems (Altlussheim, Germany) as previously described [9]. Moreover, a centromeric probe for chromosome 8 (Vysis), a subtelomeric probes for chromosome 8pter (Vysis),), a centromere-near probe in 8p11.21 (bA64C22 – BAC-PAC Chori resource) and the BAC-probes listed in Table 1 were used. The latter were kindly provided by Dr. W.W. Cai, Baylor College, Houston, Texas, USA.

**Table 1:** List of BAC probes used to confirm the presence of the duplication

**FISH-probe**	**Chromosomal Location**	**Location in MB (NCBI 36.1)**	**FISH-result**	
			
			#8	der(8)
Subtelomere probe (Vysis)	8p23	0.55	1x	1x

RP11-29A2	8p23	5.106 – 5.256	1x	2x

RP5-991O23	8p23	5.342 – 5.459	1x	2x

CTD-2629I16	8p23	6.689 – 6.785	1x	2x

RP11-540E4	8p23	8.029 – 8.179	1x	2x

RP11-211C9	8p23	8.504 – 8.677	1x	2x

RP11-241P12	8p23	9.788 – 9.958	1x	2x

RP11-177H2	8p23	10.696 – 10.796	1x	2x

RP11-589N15	8p23	11.740 – 11.803	1x	2x

RP11-433L7	8p22	14.316 – 14.461	1x	2x

RP11-60C8	8p22	15.290 – 15.445	1x	2x

RP11-44L18	8p22	15.557 – 15.699	1x	2x

RP11-255E13	8p22	16.333 – 16.472	1x	2x

RP11-19N21	8p22	16.444 – 16.618	1x	2x

RP11-525O22	8p22	17.846 – 17.950	1x	2x

bA64C22 – BAC-PAC Chori resource	8p11.21	n.a.	1x	1x

If not indicated differently the probes were derived from Dr. W.W. Cai, Baylor College, Houston, Texas, USA.

Microsatellite analysis was done as previously described [10] using the markers listed in Table 2.

**Table 2:** List of used microsatellite probes and results obtained for mother, father and child

**Marker**	**Chromosomal location**	**Location in MB (NCBI build 36.1)**	**Mother**	**Father**	**Child**	**Result**
D8S264	8p23	2.14	ac	bb	ccc	mat. UPD

D8S1099	8p23	6.04	bb	aa	bbb	mat. UPD

D8S1130	8p22	11.80	ab	bc	bb	n.i.

D8S1106	8p22	12.81	ab	ab	ab	n.i.

D8S1145	8p22	18.40	bc	ab	bbc	paternal

D8S1477	8p12	32.08	ab	cc	acc	paternal

D8S1110	8q11	53.29	bb	ab	bb	n.i.

D8S1113	8q12	59.85	ac	bd	ad	i/n

D8S1119	8q21	87.33	ab	ab	ab	n.i.

D8S1132	8q23	107.40	cc	ab	bc	i/n

D8S1128	8q24	128.65	ac	ab	bc	i/n

D8S373	8q24	143.91	bd	ac	ad	i/n

Abbreviations: a, b, c = type of alleles, mat. UPD = maternal uniparental disomy, n.i. = non informative; i.n. = informative normal.

## Results

**Figure 2:**
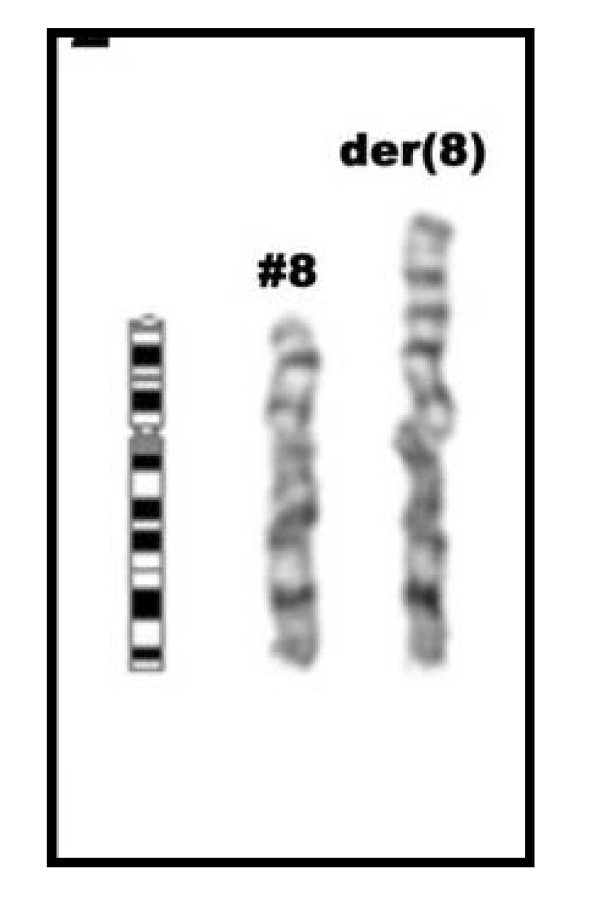
**GTG-banding result showing only the normal (#8) and the aberrant chromosome 8 of the present case, accompanied by an ideogram of a normal chromosome 8**.

**Figure 3:**
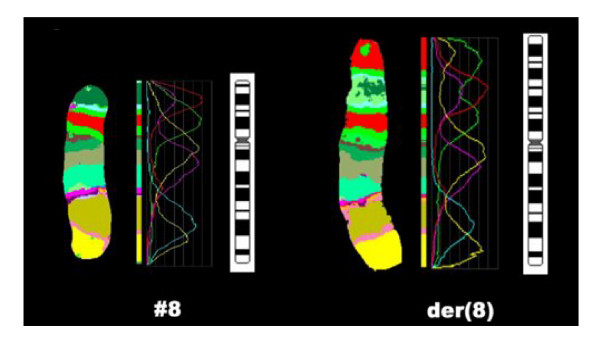
**Result of multicolor banding (MCB) shows the MCB-pseudo-coloring, the fluorochrome-profiles and the GTG-ideogram of the normal and the derivative chromosome 8**.

**Figure 4:**
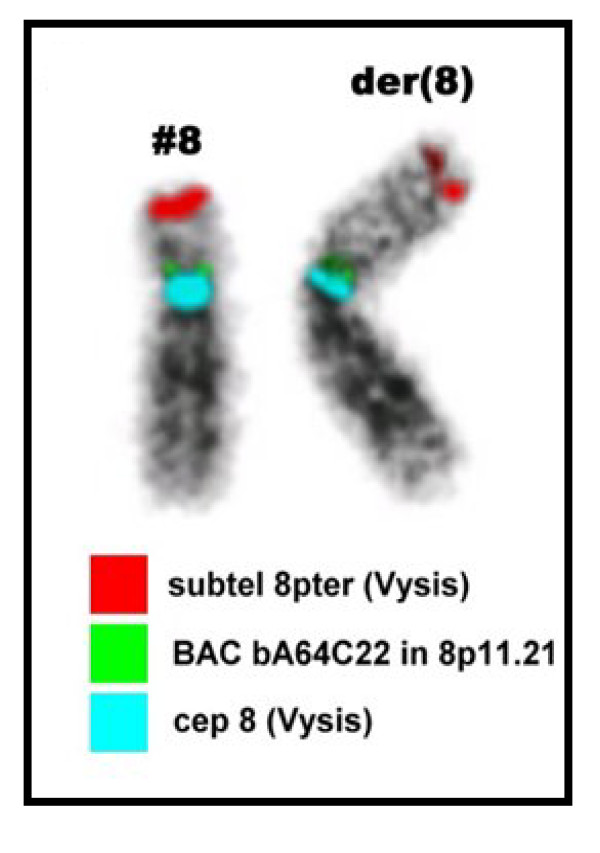
**The normal and the aberrant chromosome 8 of the present case are depicted in inverted DAPI**. A centromeric probe (blue), a centromere-near probe (green) and a subtelomeric probe (red) were hybridized together and revealed a more complex nature of the rearrangement.

GTG-banded chromosome preparations were suggestive of duplication in 8p (Figure 2). The karyotype was characterized in detail using MCB (Figure 4), subtelomeric and centromere-near probes (Figure 3). The examinations indicated an inverted duplication involving segment 8p12→8p23.3, and the karyotype was re-interpreted as 46,XY,dup(8)(pter->p23.3::p12->p23.3::p23.3->qter). The subtelomeric region on 8p was not deleted and the karyotype represented partial trisomy 8p23.3 to 8p12. Chromosome analysis of both parents revealed normal results, with no indication of a rearrangement in 8p.

**Figure 5:**
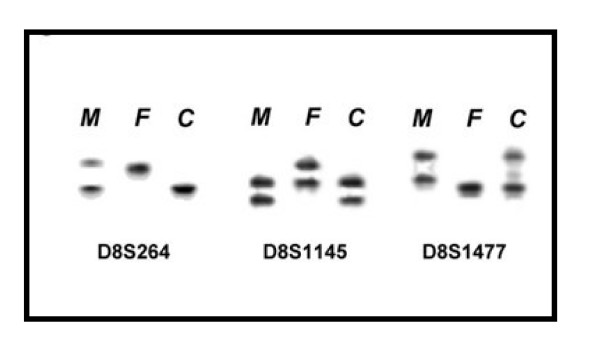
**Three examples of the microsatellite anlysis result are shown**. For the markers D8S264, D8S1145 and D8S1477 the different alleles are shown for the mother (M), the father (F) and the child (C). For result interpretation see Tab. 2.

**Figure 6:**
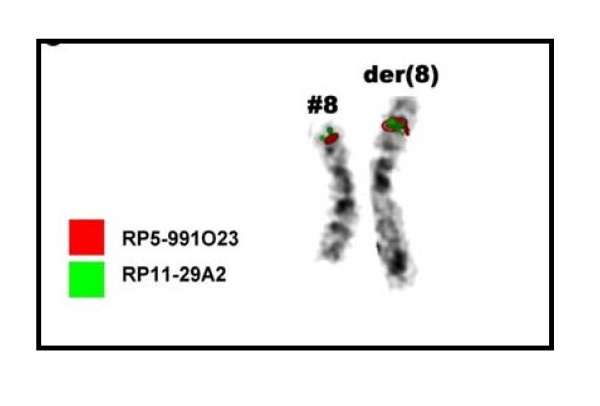
**The normal and the aberrant chromosome 8 of the present case are depicted in inverted DAPI**. In summary, the presence of three copies of the probes RP11-29A2 (green) and RP5-991O23 (red) could be proven by FISH.

**Figure 7:**
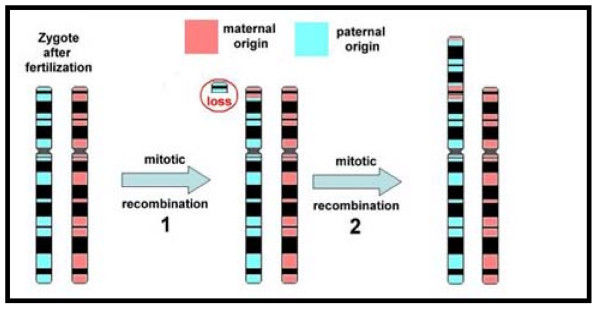
**Suggested mode of formation of the derivative chromosome 8 of the present case**.

Microsatellite analysis (Figure 6) gave hints for an inverted duplication of the paternally derived chromosome 8 (markers D8S11145 and D8S1477 in Table 2). However, only maternal alleles could be observed for the markers D8S264 and D8S1099, both located in position 2.14 and 6.04 Mb according to NCBI build 36.1. A deletion of the corresponding region could have been an explanation for this finding, however, FISH using the three probes RP11-29A2, RP5-991O23 and CTD-2629I16 located in 5.2, 5.4 and 6.6 Mb, respectively, could not confirm this possibility (see Figure 5). Thus, a mitotic recombination of maternally and paternally derived chromosomes 8, involving a loss of the paternally derived region 8p23.3 must have appeared prior to the building of the inverted duplication (Figure 7).

## Discussion

Several studies have shown that particular subset of segmental duplications such as the olfactory receptor (OR) gene clusters are the substrate for the formation of intrachromosomal rearrangements involving the short arm of chromosome 8. At the OR gene cluster, an intersister chromatid recombination [11] and an interhomologous chromatid ectopic recombination [12] event have been proposed for chromosome rearrangements of 8p. The inv dup (8) consistently originate in maternal meiosis [12] and all the mothers of subjects with inv dup (8p) are heterozygous for an inversion polymorphism, present in 26% of normal controls, between the OR gene clusters [11,12]. Furthermore, polymorphic marker analysis also indicated that inv dup (8p) was partially heterodisomic indicating that two copies of maternal allele were present [13]. In our report, microsatellite analyses revealed the paternal origin of the duplication and gave hints for a mitotic recombination involving about 6 Mb in 8p23.3. The mode of formation of the derivative chromosome 8 in the present patient was suggested as loss of paternally derived region 8p23.3 and recombination of maternally and paternally derived chromosome 8 (Figure 7)

We report on an inverted duplication of region 8p12→23.3 presenting with significant motor development delay, hypotonia, facial dysmorphisms, ventricular septal defects and corpus callosum agenesis, most of which were reported in previous studies [4-7,11]. The regions 8p21 and 8p22 were commonly duplicated in all patients with inv dup (8p). Though different breakpoint regions for inv dup (8p) are reported, the clinical findings are quite homogeneous. In our report, however, the subtelomeric region was not deleted. We propose that the phenotypic findings of these patients are mainly due to trisomy 8p12→23.3 with an inverted duplication of 8p. Recently, a duplication of 8p23.1 and triplication of 8p23.2 in patients affected by mental retardation and minor facial dysmorphisms have been presented [14].

A limited number of patients with inv dup 8p have so far been reported in the literature should not lead us to the conclusion that this duplication occurs extremely rare; it is more likely that it is rarely reported because of relatively non-specificity of the abnormalities in these patients and the cytogenetic band assignment by conventional cytogenetic analysis is difficult. The application of MCB demonstrates the occurrence of different inverted duplications within the short arm of chromosome 8.

## Conclusion

Inverted duplications on chromosome 8p are observed more frequently by the aim of technical improvement in routine cytogenetics. More complex karyotypes are being delineated by widely available use of newly developed tools. In conclusion, the present patient suggests that there might be a certain predisposition to chromosome 8p for more complex aberrations other than inverted duplications, which should be considered during the cytogenetic evaluation.

## Competing interests

The authors declare that they have no competing interests.

## Authors' contributions

DA: carried out clinical examination, cytogenetic studies, drafted the manuscript; AW: carried out MCB analysis; EU: carried out clinical evaluation; DA: carried out cardiological evaluation; KM: carried out molecular genetic studies; FVE; carried out clinical evaluation; HT: carried out MCB analysis; ET: carried out clinical evaluation; TL: carried out molecular genetic studies and drafted the manuscript.

All the authors read and approved the final manuscript.

## Consent Section

Written informed consent was obtained from the patient for publication of this case report and any accompanying images. A copy of the written consent is available for review by the Editor-in Chief of this journal.
